# Cancer-Associated Fibroblasts Promote Vascular Invasion of Hepatocellular Carcinoma via Downregulating Decorin-integrin β1 Signaling

**DOI:** 10.3389/fcell.2021.678670

**Published:** 2021-08-24

**Authors:** Xiaobo Zheng, Peng Wang, Li Li, Jing Yu, Chune Yu, Liangliang Xu, Lian Li, Fuzhen Dai, Lei Feng, Hong Zou, Xiaobo Chen, Ming Zhang, Mingqing Xu

**Affiliations:** ^1^Department of Liver Surgery, West China Hospital, Sichuan University, Chengdu, China; ^2^Institute of Clinical Pathology, West China Hospital of Sichuan University, Chengdu, China; ^3^Department of General Surgery, The First People’s Hospital of Longquanyi District, Chengdu, China; ^4^Department of Biliary Surgery, West China Hospital, Sichuan University, Chengdu, China; ^5^General Surgery Center of PLA, General Hospital of Western Theater Command, Chengdu, China; ^6^Department of Hepatopancreatobiliary Surgery, Meishan City People’s Hospital, Meishan Hospital of West China Hospital, Sichuan University, Meishan, China; ^7^Department of General Surgery, Mianzhu Hospital of West China hospital, Sichuan University, Mianzhu, China

**Keywords:** hepatocellular carcinoma, vascular invasion, portal vein tumor thrombosis, cancer-associated fibroblasts, tumor microenvironment, decorin-integrin β1 signaling

## Abstract

Hepatocellular carcinoma (HCC) is a common malignancy worldwide, and the high ratio of recurrence and metastasis remains the main cause of its poor prognosis. Vascular invasion of HCC includes microvascular invasion (MVI) and portal vein tumor thrombosis (PVTT) and is regarded as a common roadmap of intrahepatic metastasis in HCC. However, the molecular mechanism underlying vascular invasion of HCC is largely unknown. Here, we analyzed the transcriptomes of primary tumors, PVTT tissues, and tumor tissues with or without MVI. We found that extracellular matrix-related pathways were involved in vascular invasion of HCC and that decorin secreted by cancer-associated fibroblasts was gradually downregulated from normal to tumor tissues and more so in PVTT tissues. We also established that low-level decorin expression is an independent risk factor for MVI and it is associated with a poor prognosis. Decorin downregulated integrin β1 and consequently inhibited HCC cell invasion and migration *in vitro*. Co-staining DCN and integrin β1 revealed that DCN dynamically regulated integrin β1 protein expression. Integrin β1 knockdown significantly inhibited HCC invasion and migration, and decorin combined with such knockdown synergistically augmented the anti-metastatic effects. Co-IP assay confirmed the direct interaction of decorin with integrin β1. Our findings showed that targeting cancer-associated fibroblast-related decorin is not only a promising strategy for inhibiting HCC vascular invasion and metastasis but also provides insight into the clinical treatment of patients with PVTT.

## Introduction

Hepatocellular carcinoma (HCC) is a common malignancy worldwide and the fourth leading cause of cancer-related death (Bray et al., 2018). High rates of recurrence and metastasis, even after systemic treatment, comprise the main causes of a poor prognosis for patients with HCC (Forner et al., 2018). Intrahepatic metastasis derived from vascular invasion (VI) of HCC, accounts for 90% of metastases and is the primary profile of HCC metastasis (Tabrizian et al., 2015). Vascular invasion is a process of intrahepatic dissemination in which aggressive tumor cells invade blood vessels and spread to distant organs. Vascular invasion is a common phenomenon in HCC, and microvascular invasion (MVI) and portal vein tumor thrombosis (PVTT) are found in 44.0–62.2% of patients with HCC at autopsy (Lu J. et al., 2019). Vascular invasion has been regarded as an independent risk factor for a poor prognosis (Renne et al., 2020). The median overall survival (OS) of untreated patients who have HCC with PVTT is ∼ 4 months (Roayaie et al., 2009). The clinical management of such patients has been intensively investigated, and guidelines for surgical and palliative therapy have been established (Lu J. et al., 2019; Wei et al., 2019; Zhang et al., 2019). Because the mechanism of VI in HCC is not well understood, clinical treatment remains challenging. Biomarkers such as circular RNA (Fransvea et al., 2009) and imaging methods (Huang et al., 2020) have recently been applied to predict MVI, and VI in HCC has been analyzed by multi-omics (Zhang et al., 2015; Yang et al., 2017; Sulaiman et al., 2019). However, the fundamental molecular mechanism underlying VI in HCC remains largely unknown.

Tumors comprise a complex ecosystem that includes the tumor microenvironment (TME) immune cells, fibroblasts, and endothelial cells (Hernandez-Gea et al., 2013; Fu et al., 2019; Lu C. et al., 2019; Craig and von Felden, 2020). Encouraging results from recent clinical trials of therapy with immune checkpoint inhibitors have encouraged research focus on the TME (Le et al., 2015; Finn et al., 2020). Fibroblasts are a central component of the TME, and cancer-associated fibroblasts (CAFs) are involved in tumor carcinogenesis and progression. CAFs regulate tumor-initiating cell plasticity in HCC through c-Met/FRA1/HEY1 signaling (Lau et al., 2016). Peri-tumor-associated fibroblasts promote intrahepatic HCC metastasis by recruiting cancer stem cells (Jiang et al., 2017), and targeting CAFs has generated encouraging results as HCC anti-tumor therapy (Kubo et al., 2016; Lau et al., 2016; Jiang et al., 2017). However, how CAFs mediate VI to promote HCC metastasis remains poorly understood.

Decorin (DCN) is a prototypical small leucine-rich proteoglycan and important component of the cellular microenvironment or extracellular matrix (ECM) (Feugaing et al., 2013). The *DCN* gene is a marker of fibroblasts and is most commonly distributed in fibroblasts (Neill et al., 2016). Its interactions with matrix and cell membrane components have been implicated in many physiological and pathophysiological processes, including matrix organization, signal transduction, wound healing, cell migration, inhibition of metastasis, and angiogenesis (Järveläinen et al., 2015). Decorin binds with high affinity to various receptor tyrosine kinases including Met, EGFR, IGF-IR, PDGFR, and VEGFR2, to induce a multitude of oncosuppressive functions, including the inhibition of tumor growth and progression (Bi et al., 2008; Horváth et al., 2014). Decorin also acts as a pro-inflammatory agent by modulating macrophage function and cytokine secretion (Jármay et al., 2000). Therefore, DCN is an ideal therapeutic candidate for controlling solid malignancies. However, how DCN regulates VI in HCC remains unclear.

We analyzed the transcriptomes of primary tumor and PVTT tissues from patients with HCC, as well as tumor tissues with or without MVI. We established that ECM-related pathways mediated VI by HCC, and that DCN gradually became downregulated from normal to tumor and further in PVTT tissues. We found that DCN was mainly expressed in fibroblasts, indicating that these cells promoted VI by HCC by regulating DCN secretion. We also found that DCN expression in tumor tissues was associated with MVI, and that low DCN expression was associated with a poor prognosis. Decorin inhibited the invasion and migration of HCC by downregulating integrin β1 *in vitro*.

## Materials and Methods

### Data Acquisition

We obtained data about patients with HCC tumors, PVTT tissues, and mRNA sequences from GSE77509 in the Gene Expression Omnibus (GEO) database. The HCC tumor tissues with MVI and without the MVI mRNA sequencing set and patient personal information and clinical pathological features were obtained from The Cancer Genome Atlas (TCGA) database^1^. For further verification, we downloaded independent microarray datasets (GSE69164, GSE74656) from GEO. According to the publication guidelines, the datasets can be used for publication without restriction or limitation^2,^^3^.

### Patients and Specimens

Paired normal, tumor and PVTT tissues were collected from patients with HCC at West China Hospital, Sichuan University, Chengdu, China. Detailed clinicopathological parameters for each patient were extracted from the digital health care system of West China Hospital. The Biomedical Ethics Committee of West China Hospital approved the study protocols, and all patients signed written, informed consent forms.

### Cell Culture

We maintained the HCCLM3, HEK293T, and Hep3B cells (Cell Bank of Type Culture Collection, Chinese Academy of Sciences, Shanghai, China) maintained in Dulbecco’s modified Eagle medium (DMEM)/high glucose medium (Hyclone, Logan, UT, United States) supplemented with 10% fetal bovine serum (FBS) (PAN-Biotek, Aidenbach, Bavaria) and 1% penicillin-streptomycin (Hyclone) at 37°C in a humidified 5% CO_2_ atmosphere. The authenticity of the cell line was verified by DNA fingerprinting before use. We explored the function of 1 and 4 μg/mL of polypeptide DCN (R&D Systems, Minneapolis, MN, United States) in HCC HCCLM3 and Hep3B cells *in vitro* in the above medium. Blank medium was the control.

### RNA Extraction and Quantitative Real-Time PCR (qRT-PCR)

Total RNA was extracted from each specimen using Trizol (Invitrogen, Carlsbad, CA, United States) as described by the manufacturer. The concentration and quality of RNA were assessed by measuring absorbance ratios of A260/A280 and A260/A280 using a ScanDrop Nuclear Acid Analyzer (Analytik Jena GmbH, Jena, Germany). Complementary DNAs (cDNAs) were generated using Reverse Transcription System Kits (Vazyme Biotech Co., Ltd., Nanjing, China), and amplified by qRT-PCR in triplicate using Maxima SYBR Green qPCR Master Mix (Vazyme) on a CFX connect real-time system (Bio-Rad, Hercules, CA, United States) as described by the manufacturer. The glyceraldehyde 3-phosphate dehydrogenase (GAPDH) gene was the internal control for each gene. Relative expression levels of each gene were calculated using the 2^–ΔΔCt^ method. We determined ΔCt by subtracting the Ct of GAPDH mRNA from that of each gene. Table 1 shows the qRT-PCR primers.

**TABLE 1:** Primers used in our study.

Primer name	Forward	Reverse
DCN	CAGTGTTCTGATTTGGGTCT	CCATCTTTGATTTCGGTTAT
COL1A1	GAGGGCCAAGACGAAGACATC	CAGATCACGTCATCGCACAAC
COL3A1	GGAGCTGGCTACTTCTCGC	GGGAACATCCTCCTTCAACAG
COL4A1	GGGATGCTGTTGAAAGGTGAA	GGTGGTCCGGTAAATCCTGG
Fibronectin 1	GAGAATAAGCTGTACCATCGCAA	CGACCACATAGGAAGTCCCAG
Integrin α5	GCCTGTGGAGTACAAGTCCTT	AATTCGGGTGAAGTTATCTGTGG
Integrin β3	AGTAACCTGCGGATTGGCTTC	GTCACCTGGTCAGTTAGCGT
GAPDH	ACTCCTCCACCTTTGACGC	GCTGTAGCCAAATTCGTTGTC

**qPCR quantitative polymerase chain reaction, DCN decorin.**

### Integrin β1 Lentivirus shRNA Constructs

The core sequence for constructing an shRNA plasmid targeting integrin β1 was 5′- GCCTTGCATTACTGCTGATAT-3′. Lentivirus preparations were produced by co-transfecting the helper virus packaging plasmids pMD2.G, psPAX2, and pLKO.1 puro (empty vector or containing shRNA) into HEK293T cells. Supernatants containing viruses were collected after 48-h incubation.

### Protein Isolation and Western Blotting

Total protein was lysed in RIPA buffer (Beyotime Biotechnology, Shanghai, China) containing 1% protease inhibitor (Cell Signaling Technology, Danvers, MA, United States). Proteins in lysates were quantified using Pierce bicinchoninic acid (BCA) protein assay kits (Beyotime Biotechnology), and then 30 μg were separated by 10% SDS-PAGE and transferred to polyvinylidene fluoride (PVDF) membranes. Non-specific protein binding was blocked with 5% non-fat dry milk in Tris buffered saline-Tween (TBST) for at least 1 h, then the membranes were incubated at 4°C overnight with the following primary antibodies diluted 1:1,000 unless otherwise stated: DCN (ab175404), integrin α1 (ab243032), integrin α3 (ab242196), integrin α11 (ab198826, 1:800 dilution; all from Abcam, Cambridge, United Kingdom), vimentin (5741S), N-cadherin (13116S), β-catenin (8480S), E-cadherin (3195S), HER2/ErbB2 (4290S), integrin β5 (3629S), integrin β1 (34971S; all from Cell Signaling Technology), TGF beta-1 (MA5-16949), and TGF beta-2 (710276; diluted 1:750, both from Thermo Fisher Scientific Inc., Waltham, MA, United States), MMP2 (10373-2-AP undiluted; Proteintech, Group Inc., Rosemont, IL, United States) and GAPDH (200306-7E4 diluted 1:2,000; Zen BioScience, China). The membranes were then incubated with secondary antibody diluted 1:5,000 (Zenbio, Chengdu, China) at 37°C for 1 h and immersed in SuperSignal West Femto Agent (Millipore Sigma Co., Ltd., Burlington, MA, United States). Protein signals were detected by the Chemical Mp Imaging System (Bio-Rad) and proteins were quantified using ImageJ. The internal reference was GAPDH.

### Immunofluorescence Assays

Normal, tumor, and PVTT tissues were fixed in 4% paraformaldehyde, embedded in paraffin, and cut into 4-μm sections. After three washes with PBS, non-specific protein binding was blocked with 5% bovine serum albumin (BSA) at room temperature for 1 h. The sections were incubated at 4°C overnight with anti-DCN diluted 1:200, and anti-E-cadherin, anti-αSMA, and anti-integrin β1 all diluted 1:100. The sections were incubated on the following day with a 1:500-diluted secondary antibody labeled with a fluorescent dye (Life Technologies Corporation, Carlsbad, CA, United States) at 37°C for 40 min and stained with DAPI for 10 min at room temperature. Stained cells were visualized by fluorescence microscopy (Leica, Mannheim, Germany) or Nikon N-STORM confocal microscopy (Nikon Corp., Tokyo, Japan).

### Immunoprecipitation (IP)

Cells on ice were lysed using a buffer provided with Co-IP kits containing protease inhibitors (abs955; Absin Bioscience Inc., Shanghai, China), as described by the manufacturer. Lysates were centrifuged at 14,000 × g at 4°C for 10 min, and then the soluble fraction was clarified by incubation with protein A/G agarose beads. Proteins in the cleared supernatant were immunoprecipitated using the indicated primary antibodies at 4°C overnight, then incubated with Protein A/G beads at 4°C for 12 h. The immunoprecipitated complexes were rinsed and western blotted. The positive control was Input.

### Wound Healing Assays

Cells cultured in 6-well plates were scratched using a sterilized pipet tip, gently rinsed with PBS, then in DMEM/high glucose medium containing 0.5% FBS and 1% penicillin/streptomycin. Images were acquired using an Olympus digital camera every 24 h.

### Transwell Assays

Cells were suspended in 300 μL of serum-free DMEM medium, and placed in the upper chamber of 24-well Transwell chambers (MilliporeSigma Co., Ltd., Burlington, MA, United States) coated with Matrigel with 8-μm pores (BD Biosciences, San Jose, CA, United States). Chemoattractant medium containing 10% FBS was placed in the lower chamber. Cells that did not penetrate the matrix after 48 h were removed. The inserts were then visualized by staining with 0.2% crystal violet and counted using an inverted microscope.

### Statistical Analysis

Data were statistically analyzed using GraphPad Prism 8 (GraphPad Software, San Diego, CA, United States) and SPSS version 25.0 (IBM Inc., Armonk, NY, United States). Normally distributed data are presented as means ± standard deviation (SD). If the 95% confidence interval (CI) did not include the value 1, then values with *P* < 0.05 were considered statistically significant. Differences between datasets were assessed using one-way ANOVA and two-tailed Student *t*-tests. The cut-off value was the median expression of DCN. Risk factors associated with MVI were identified by univariate and multivariate binary logistic regression analyses. Kaplan-Meier survival curves were plotted and survival was compared using log-rank tests.

## Results

### Pathways Associated With ECM Are Involved in VI by HCC

**FIGURE 1:**
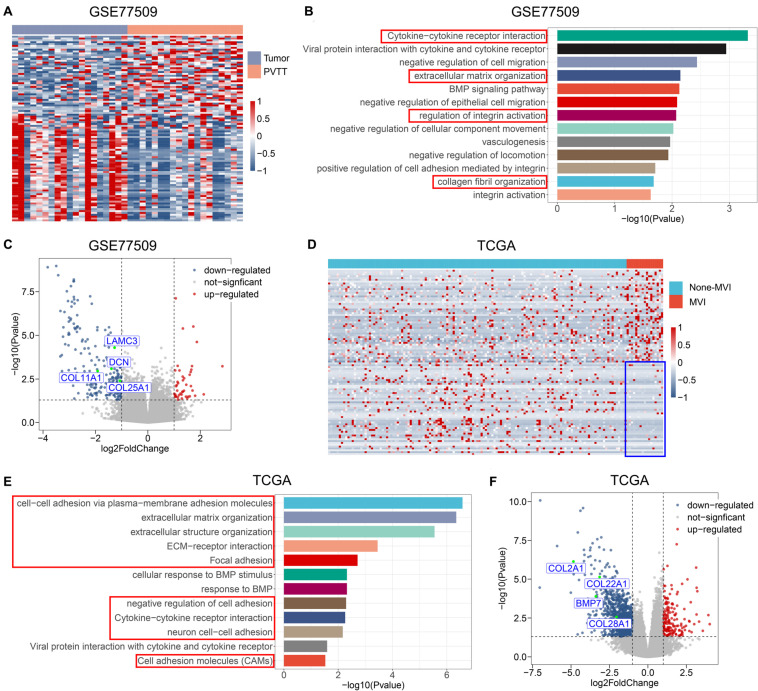
ECM-related pathways and genes are involved in vascular invasion of HCC. **(A)** Heatmap of top 20 DEGs between tumor tissues and PVTT tissues from public dataset GSE77509; the colors represent Z-Score of log-normalized data. **(B)** The significantly altered molecular pathways between tumor tissues and PVTT tissues from public dataset GSE77509. Red frame indicates ECM-related pathways. **(C)** Volcano plot of DEGs in tumor tissues and PVTT tissues from public dataset GSE77509; multiple ECM-associated genes are shown. **(D)** Heatmap of top 20 DEGs between none-MVI tumor tissues and MVI tumor tissues from TCGA; the colors represent Z-Score of log-normalized data. Blue frame indicates consistent lower expression of top 20 downregulated genes in MVI group across patients. **(E)** The significantly altered molecular pathways between none-MVI tumor tissues and MVI tumor tissues from TCGA. Red frame indicates ECM-related pathways. **(F)** Volcano plot of DEGs in none-MVI tumor tissues and MVI tumor tissues from TCGA, and multiple ECM-associated genes are represented. MVI, microvascular invasion; TCGA, The Cancer Genome Atlas.

We analyzed changes in the transcriptomes of malignant cells during VI to understand the molecular mechanism of VI by HCC. Clinical samples of PVTT are available as this is a common stage of macrovascular invasion by HCC. Therefore, we compared the transcriptome of primary tumor samples with that of PVTT tissues. We reanalyzed the RNA-seq data of 20-paired primary tumor and PVTT tissues from the GEO GSE77509 datasets. Transcript profiles varied between primary tumor and PVTT tissues, and numerous differentially expressed genes (DEGs) were identified between these tissues (Figure 1A). We analyzed Gene Ontology (GO)/Kyoto Encyclopedia of Genes and Genomes (KEGG) enrichment of these DEGs to identify which molecular signatures were enriched. The significantly enriched ECM-related pathways were cytokine-cytokine receptor interaction, ECM organization, regulation of integrin activation, and collagen fibril organization (Figure 1B). The ECM-related genes, *DCN*, *COL11A1*, *LAMC3*, and *COL25A1*, were also significantly downregulated in PVTT, compared with primary tumor tissues (Figure 1C), indicating that the ECM is involved in macrovascular invasion by HCC.

To confirm that the ECM is involved in the formation of MVI, we compared transcriptomic alterations between tumor tissues with and without MVI derived from patients with TNM stage I HCC from TCGA. The DEGs that were significantly upregulated in the group with MVI compared with the group without MVI were not exclusively upregulated in PVTT tissues, possibly because of the high degree of tumor heterogeneity among patients (Figure 1D). Notably, the expression of DEGs that were significantly downregulated in the group with MVI relative to that without MVI was consistently lower across patients with MVI. These findings indicated that these genes play fundamental roles in regulating MVI development (Figure 1D). The downregulated genes in the MVI group were significantly enriched in ECM-related pathways (Figure 1E), which agreed with the dysregulated pathways between the primary tumor and PVTT samples (Figure 1B). Similarly, the ECM-related genes *BMP7*, *COL2A1*, *COL22A1*, and *COL28A1*, were significantly downregulated in the group with MVI (Figure 1F). Collectively, these results indicated that the downregulation of ECM-related pathways is an important molecular event mediating the entire process of VI, from MVI to macrovascular invasion.

### Downregulation of DCN Secreted by CAFs Facilitated VI by HCC

**FIGURE 2:**
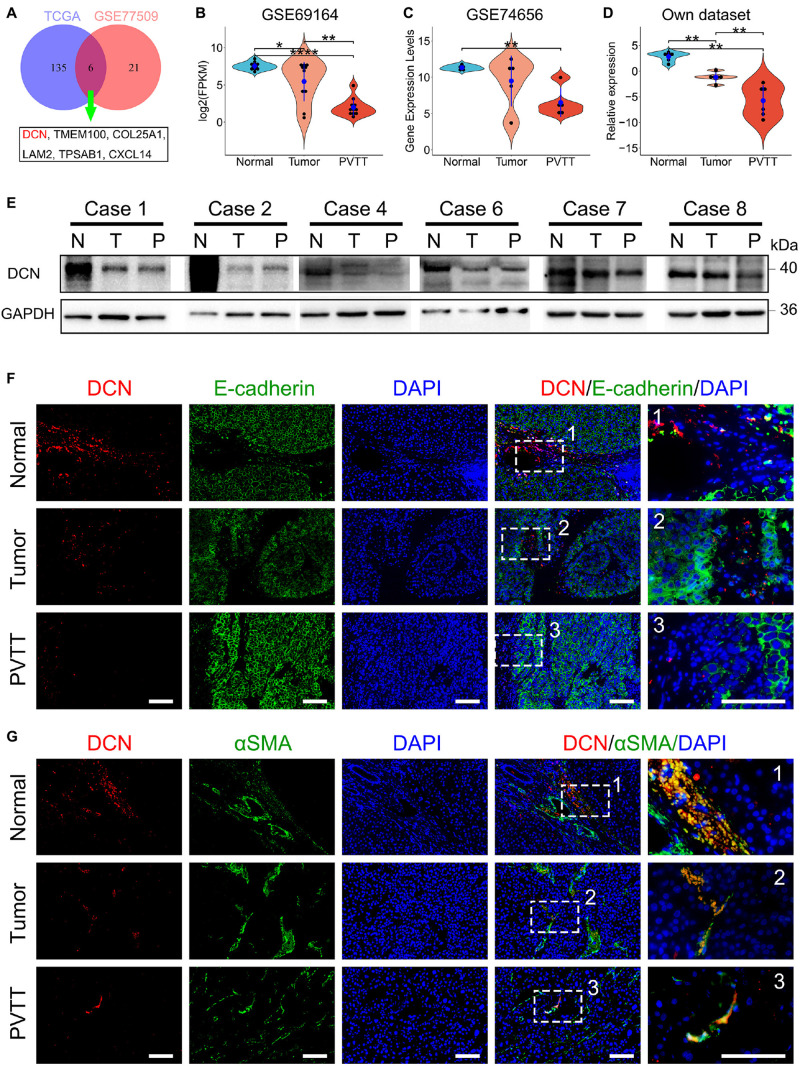
DCN secreted by CAFs is gradually downregulated during HCC progression. **(A)** Venn diagram of DEGs enriched in ECM-related pathways between public dataset GSE77509 and TCGA. **(B–D)** Relative mRNA expression of DCN among normal tissue, tumor tissue, and PVTT tissue in the GEO datasets GSE69164 **(B)**, GSE74656 **(C)**, and our dataset **(D)**. **(E)** DCN protein expression in normal tissue (N), tumor tissue (T), and PVTT (P) tissue as obtained by immunoblot analysis. **(F,G)** Co-immunofluorescence staining of DCN with E-cadherin **(F)** and DCN with α-SMA **(G)** was performed in normal tissue, tumor tissue, and PVTT tissue. Scale bar, 100 μm. α-SMA, alpha-smooth muscle actin; PVTT, portal vein tumor thrombosis. TCGA, The Cancer Genome Atlas. Data presented as mean ± SEM. *P < 0.05, **P < 0.01, and ****P < 0.0001, Student’s *t*-test.

We analyzed DEGs that were involved in the ECM pathway to identify potential molecules that downregulate ECM pathways and promote VI by HCC. We analyzed intersects of the DEGs enriched in the ECM pathway (Figures 1B,E) between GSE77509 and TCGA cohorts to identify common dysregulated genes. A Venn diagram revealed that the ECM-related genes, *DCN*, *TMEM100*, *COL25A1*, *LAM2*, *TPSAB1*, and *CXCL14*, were simultaneously dysregulated in both cohorts (Figure 2A). To confirm the recurrent downregulation of these genes during VI by HCC, we analyzed the mRNA expression levels of these genes in normal, tumor, and PVTT tissues from internal and external cohorts. We found that DCN gradually decreased during progress from normal to primary tumor and metastatic tissues from the public GSE69164 dataset and in our cohort (Figures 2B,D). The expression of DCN was significantly downregulated in PVTT, compared with normal tissues in the public GSE74656 dataset. Although less DCN was expressed in PVTT, than in tumor tissues in GSE74656, the values did not reach statistical significance because of the small sample size (Figure 2C). These results confirmed that DCN expression was downregulated in tumor tissues and further downregulated in PVTT tissues at the mRNA level, suggesting that DCN plays anti-tumorigenic and anti-metastatic roles.

We then analyzed DCN expression at the protein level in clinical tissues from our cohort. Immunoblotting findings showed that DCN gradually decreased from normal tissue adjacent to tumors, to primary tumors and more so in PVTT tissues (Figure 2E and Supplementary Figure 1). To confirm the subcellular location of DCN, we immunohistochemically co-stained E-cadherin with DCN and alpha-smooth muscle actin (a-SMA) in formalin-fixed paraffin-embedded samples. a-SMA is considered as the main marker of fibroblasts in numerous cancers (Lau et al., 2016; Jiang et al., 2017). The finding that DCN co-localized with a-SMA but not E-cadherin indicated that DCN is preferentially expressed in fibroblasts and not in epithelial cells (Figures 2F,G). This was consistent with the finding that DCN secreted by fibroblasts is a matrix-mediating agent in cancer development (Neill et al., 2016). In addition, DCN was gradually downregulated from normal fibroblasts to primary tumor-associated fibroblasts and further in PVTT-associated fibroblasts (Figure 2G). These results indicated that downregulating DCN secreted by fibroblasts promotes VI of HCC.

### Low DCN Expression Is Associated With MVI Occurrence and Poor Prognosis

**FIGURE 3:**
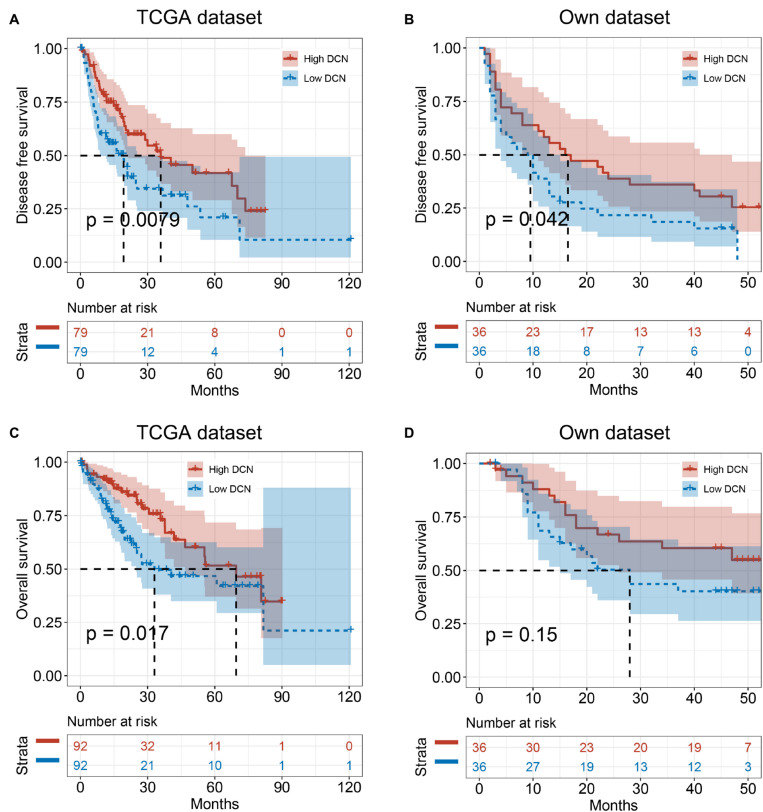
Low DCN expression levels correlate with poor prognosis. **(A,B)** Kaplan–Meier analyses showing the correlations between DCN expression level and disease-free survival of patients with HCC from TCGA database **(A)** and our own dataset **(B)**. **(C,D)** Kaplan–Meier analyses of the correlations between DCN expression level and overall survival of patients with HCC from TCGA database **(C)**, and our own dataset **(D)**. The median expression level was used as the cut-off. Values are expressed as the median with interquartile range. TCGA, The Cancer Genome Atlas.

We analyzed correlations between DCN mRNA expression in HCC tumor tissues and clinical pathological characteristics to determine the clinical importance of DCN expression. Tumor DCN levels in our dataset significantly differed only between subgroups of patients divided by MVI status (yes or no, *p* = 0.048; Table 2). We explored potential risk factors for MVI to confirm this correlation between DCN expression and MVI. Univariate analysis revealed that various characteristics, including age (<60 years), Ishak grade (≥6), incomplete tumor capsule, and low tumor DCN expression, were risk factors for MVI (Table 3). Moreover, multivariate logistic models showed that low tumor DCN expression was an independent risk factor for MVI. Kaplan-Meier findings significantly associated decreased DCN expression with shorter disease-free survival in both the TCGA and our dataset (Figures 3A,B). These results agreed with the reduced DCN levels that were associated with MVI. This might be because a lower abundance of DCN facilitates the development of VI, which accelerates tumor recurrence and metastasis. In addition, Kaplan-Meier analysis significantly associated decreased DCN expression with shorter OS in the TCGA dataset (Figure 3C). Similarly, low DCN expression positively correlated with poor OS in our dataset, although the correlation did not reach statistical significance (Figure 3D). Collectively, these results suggested that DCN is involved in the formation of MVI in patients and could serve as a potential prognostic indicator for patients with HCC.

**TABLE 2:** Relationship between the expression of decorin in tumor tissues and clinical characteristics of HCC patients.

Clinical parameters	Patient number (total = 73)	log_2_DCN relative expression	
		**Mean ± SD**	***P*-value**
Gender			0.085
Female	12	1.10 ± 2.14	
Male	61	2.56 ± 4.22	
Age			0.054
< 60	58	1.91 ± 4.06	
≥ 60	15	3.93 ± 3.28	
AFP			0.626
< 400	40	2.53 ± 4.10	
≥ 400	33	2.07 ± 3.87	
HbsAg			0.293
Positive	70	2.39 ± 4.03	
Negative	3	0.68 ± 2.12	
HBV DNA			0.927
Positive	61	2.37 ± 4.07	
Negative	11	2.25 ± 3.75	
Cirrhosis			0.128
Yes	26	1.34 ± 4.45	
No	41	2.95 ± 3.58	
Tumor number			0.391
Single	49	2.61 ± 3.95	
Multiple	24	1.74 ± 4.06	
Tumor size			0.453
≤ 5	25	2.81 ± 3.99	
> 5	48	2.07 ± 3.99	
BCLC stage			0.497
A + B	49	2.09 ± 3.85	
C	24	2.80 ± 4.26	
Tumor capsule			0.26
Complete	28	1.69 ± 3.97	
Infiltration	42	2.79 ± 3.99	
Satellite lesions			0.594
Yes	13	2.95 ± 4.74	
No	60	2.19 ± 3.83	
GVI			0.878
Yes	21	2.42 ± 4.58	
No	50	2.24 ± 3.81	
MVI			0.048*
Yes	33	1.33 ± 3.35	
No	40	3.14 ± 4.30	
Differentiation			0.719
High + moderate	40	2.48 ± 4.11	
Moderate + low	33	2.14 ± 3.87	

AFP, alpha feto protein; BCLC, Barcelona-Clinic Liver Cancer; GVI, gross vascular invasion (defined as tumor embolus in first or second branches of portal veins found by preoperative CT or MRI); HBsAg, hepatitis B surface antigen; MVI, microvascular invasion, SD standard deviation. *Statistically significant.

**TABLE 3:** Univariate and multivariate analysis of risk factors of MVI.

Factors	Regression coefficient β	OR (95% CI)	*P*-value
**Univariate analysis**			
Gender (male vs. female)	−0.331	0.718 (0.480–1.076)	0.108
Age (year ≥ 60 vs. < 60)	−0.981	0.375 (0.147–0.958)	0.040*
Tumor diameter (cm ≥ 5 vs. < 5)	−0.279	0.757 (0.463–1.236)	0.266
Tumor number (multiple vs. single)	0.405	1.500 (0.674–3.339)	0.321
Ishak grade (≥ 6 vs. < 6)	−0.821	0.440 (0.217–0.894)	0.023*
GVI (presence vs. absence)	0.511	1.667 (0.729–3.808)	0.226
Satellite nodules (presence vs. absence)	0.288	1.333 (0.463–3.843)	0.594
Tumor capsule (complete vs. incomplete)	−0.811	0.444 (0.225–0.877)	0.019*
BCLC stage (B + C vs. 0 + A)	0.693	2.000 (0.899–4.452)	0.090
AFP (ng/ml ≥ 400 vs. < 400)	0.095	1.100 (0.600–2.015)	0.758
Edmondson-Steiner grade (I/II vs. III/IV)	−0.463	0.630 (0.343–1.155)	0.135
HBV-DNA (copies ≥ 10^3^ vs. < 10^3^)	−0.492	0.611 (0.289–1.294)	0.198
HBsAg (positive vs. negative)	−0.340	0.712 (0.467–1.085)	0.114
Tumor DCN expression (high vs. low)	−0.916	0.400(0.215–0.743)	0.004**
**Multivariate analysis**			
Age (year ≥ 60 vs.**< 60)**	−0.467	0.627 (0.206–1.908)	0.411
Tumor capsule (complete vs. incomplete)	−0.382	0.682 (0.321–1.449)	0.320
Tumor DCN expression (high vs. low)	−0.831	0.436 (0.211–0.900)	0.025*

Tumor DCN expression is independent risk factors of MVI. OR odds ratio, CI confidence interval, GVI gross vascular invasion, BCLC Barcelona-Clinic Liver Cancer, AFP alpha-fetoprotein, HBV-DNA hepatitis B deoxyribonucleic acid, HBsAg hepatitis B surface antigen, DCN decorin. *A significant correlation was detected at P < 0.05, **P < 0.01.

### Decorin Inhibited HCC Cell Migration and Invasion *in vitro*

**FIGURE 4:**
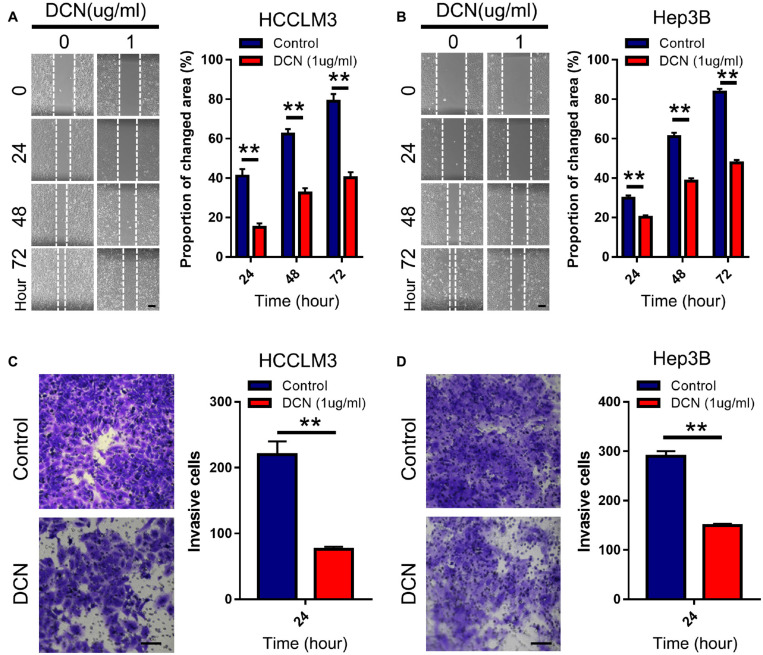
DCN inhibits migration and invasion of HCC cell lines. **(A,B)** Wound-healing assays for HCCLM3 **(A)**, and Hep3B **(B)** cells treated with DCN (1 μg/mL) or the negative control. Scale bars, 200 μm. **(C,D)** Transwell assays for HCCLM3 **(C)**, and Hep3B **(D)** cells treated with DCN(1 μg/mL) or the negative control. Scale bars, 100 μm. Data presented as mean ± SEM. **P < 0.01, Student’s *t*-test.

We evaluated the effects of DCN on HCC cell migration and invasion to functionally validate the biological role of DCN in HCC metastasis. Considering that DCN affects tumor cells mainly via extracellular signaling, we added DCN to culture medium as ectopic expression. Notably, adding 1 μg/mL DCN to the culture medium significantly inhibited HCCLM3 and Hep3B cell migration (Figures 4A,B) and invasion (Figures 4C,D). These results confirmed the anti-metastatic function of DCN in HCC cells.

### Decorin Downregulated Integrin β1 Expression in HCC

To explore the downstream targets of DCN involved in inhibiting HCC metastasis, we analyzed proteins related to the epithelial mesenchymal transition (EMT) that are involved in tumor metastasis (Yang et al., 2020). Levels of E-cadherin were high in normal tissues but decreased in tumor and PVTT tissues among our clinical samples. The expression of N-cadherin and vimentin, that are markers of mesenchymal cells, was upregulated in tumor and PVTT tissues (Supplementary Figures 2A,B). These results confirmed that tumor cells initiated the EMT to promote tumorigenesis and metastasis. However, N-cadherin, vimentin, and MMP2 expression did not significantly differ between 1 and 4 μg/mL DCN and controls, indicating that the EMT and MMP2 are not regulated by DCN (Supplementary Figures 2C,D). We then investigated the expression of TGF-β1, TGF-β2, and receptor tyrosine kinases (HER2), which are signaling molecules involved in DCN-mediated tumor carcinogenesis and metastasis (Hildebrand et al., 1994; Goldoni et al., 2008). The expression of HER2, TGF-β1, and TGF-β2 was slightly decreased in the DCN, compared with control HCCLM3 and Hep3B cells (Supplementary Figures 2E,F). These results are consistent with those of previous studies (Hildebrand et al., 1994; Goldoni et al., 2008), and validated accuracy of our findings.

**FIGURE 5:**
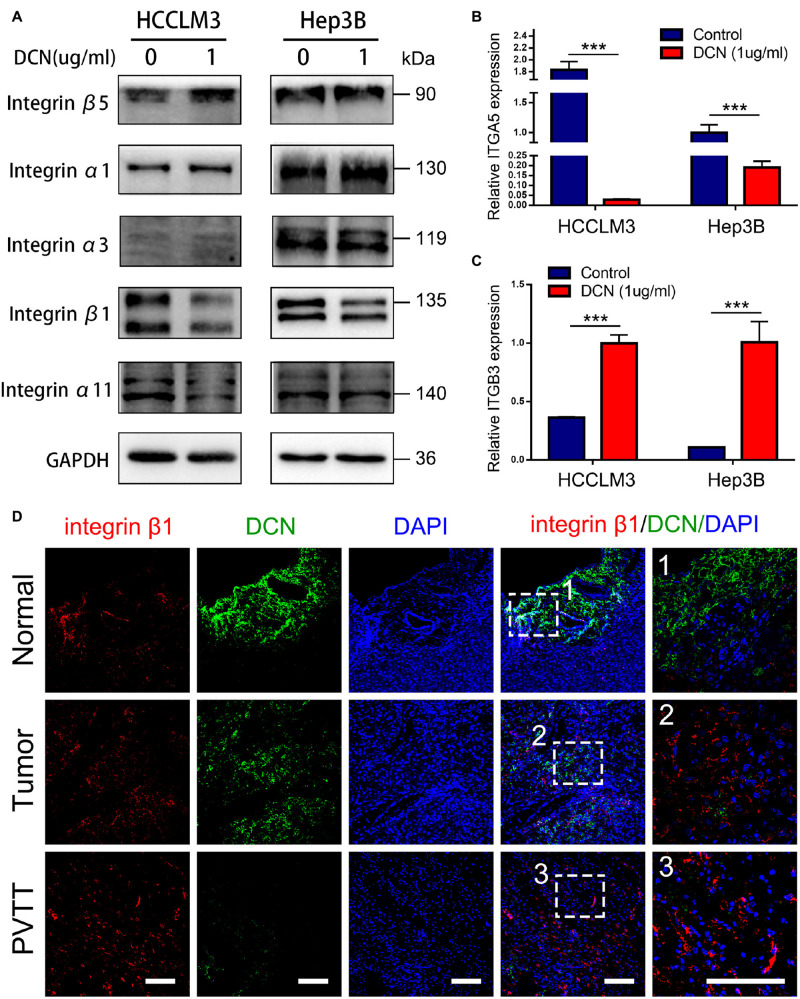
DCN downregulates integrin β1 expression. **(A)** Western blot analysis of integrins expression in HCCLM3 and Hep3B cells treated with DCN (1 μg/mL) or the negative control. **(B)** qPCR analysis of integrin α5 expression in HCCLM3 and Hep3B cells treated with DCN (1 μg/mL) or the negative control. **(C)** qPCR analysis of integrin β3 expression in HCCLM3 and Hep3B cells treated with DCN (1 μg/mL) or the negative control. **(D)** Representative images of integrin β1 and DCN expressions in normal tissue, tumor tissue, and PVTT tissue obtained by co-immunofluorescence staining. Scale bar, 100 μm. ITGA5, integrin α5; ITGB3, integrin β3; PVTT, portal vein tumor thrombosis. Data presented as mean ± SEM. ***P < 0.001, Student’s *t*-test.

Integrins are involved in tumor progression and drug resistance (Hamidi and Ivaska, 2018; Yu et al., 2020). As shown by the transcriptomic results of clinically matched samples from patients who had HCC with PVTT, signals of ECM organization and regulation of integrin activation were enriched during PVTT development (Figure 1B). To confirm whether DCN inhibits HCC metastasis through regulating the integrin pathway, we analyzed the expression of integrins α1, α3, α11, β1, and β5 in DCN and control HCCLM3 and Hep3B cells at the protein level. The expression of integrins β1 and α11 was significantly downregulated in response to enhanced DCN expression, whereas that of integrins α1, α3, and β5 was not changed, suggesting that DCN binds to integrin β1 or integrin α11 to inhibit HCC metastasis (Figure 5A and Supplementary Figure 3). We also analyzed the expression of integrins α5 and β3 at the mRNA level in the DCN and control groups. The expression of integrin α5 was downregulated by upregulated DCN, whereas integrin β3 was upregulated by enhancing DCN expression in HCCLM3 and Hep3B HCC cells (Figures 5B,C). The ECM components COL1A1, COL3A1, COL4A1, and fibronectin 1 (FN1), are critical regulators during tumor metastasis, so we examined their expression in the DCN and control cells using qPCR. The upregulation of DCN resulted in downregulated COL1A1, and upregulated FN1 expression (Supplementary Figure 4).

To confirm that integrin β1 is a critical factor in VI and HCC metastasis, we analyzed integrin β1 protein expression in clinical samples. We found significantly upregulated integrin β1 protein expression in epithelial cells of primary tumor and PVTT, compared with normal tissues (Supplementary Figure 5). More importantly, co-staining DCN and integrin β1 revealed that DCN dynamically regulated integrin β1 protein expression, in that a decrease in DCN was accompanied by integrin β1 upregulation from normal, to primary tumor and PVTT tissues (Figure 5D). Collectively, these results suggest that DCN regulates integrin β1 to promote HCC metastasis.

### Decorin Plays Anti-metastatic Role in HCC by Binding to Integrin β1

**FIGURE 6:**
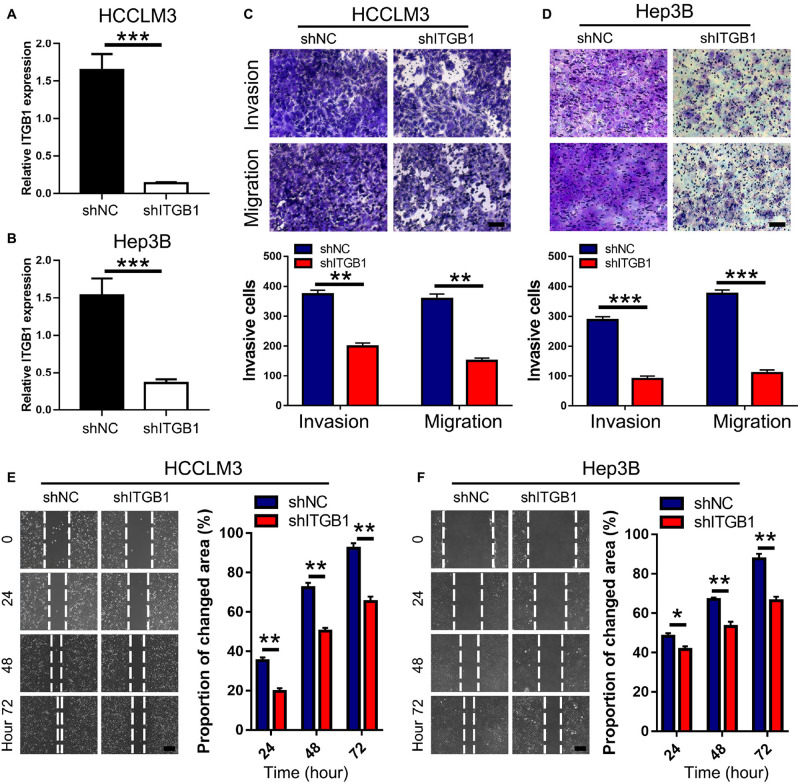
Integrin β1 downregulation inhibits migration and invasion of HCC cell lines. **(A,B)** Results of qRT-PCR validated the integrin β1 knockdown in HCCLM3 **(A)** and Hep3B **(B)** cells. **(C,D)** Transwell assays for HCCLM3 **(C)**, and Hep3B **(D)** cells transfected with integrin β1 specific shRNA or the negative control. Scale bars, 100 μm. **(E,F)** Wound-healing assays for HCCLM3 **(E)**, and Hep3B **(F)** cells transfected with integrin β1 specific shRNA or the negative control. Scale bars, 200 μm. ITGB1, integrin β1. Data presented as mean ± SEM. *P < 0.05, **P < 0.01, and ***P < 0.001, Student’s *t*-test.

To confirm the pro-metastatic role of integrin β1 in HCC, we evaluated the effects of integrin β1 knockdown on HCCLM3 and Hep3B cell migration and invasion. We downregulated integrin β1 expression in these cell lines using shRNA and confirmed the knockdown by qPCR (Figures 6A,B). Notably, integrin β1 knockdown significantly inhibited HCCLM3 and Hep3B cell migration and invasion in Transwell chambers (Figures 6C,D). The results of wound healing assays further confirmed that integrin β1 downregulation significantly inhibited HCCLM3 and Hep3B cell migration (Figures 6E,F). These results confirmed the pro-metastatic role of integrin β1 in HCC.

**FIGURE 7:**
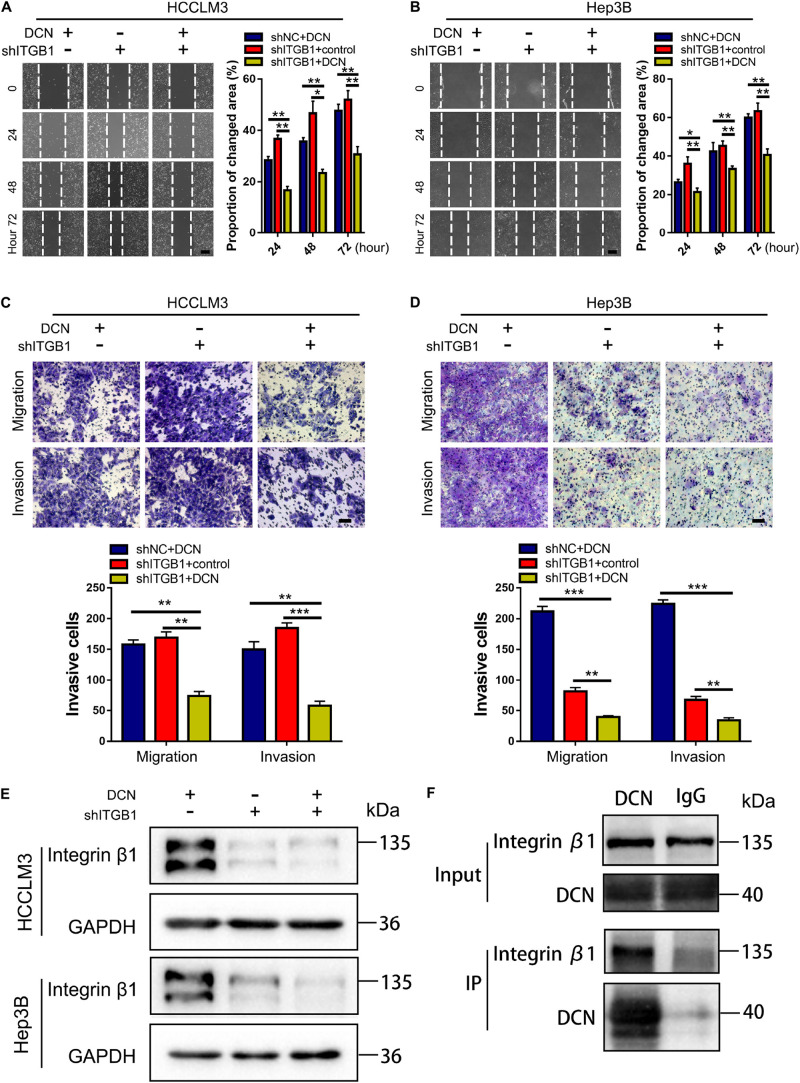
DCN treatment combined with integrin β1 downregulation synergistically inhibits migration and invasion of HCC cell lines. **(A,B)** Wound-healing assays using integrin β1 knocked-down or negative control HCCLM3 **(A)**, and Hep3B **(B)** cells treated with DCN (1 μg/mL) or the negative control. Scale bars, 200 μm. **(C,D)** Transwell assays using integrin β1 knocked-down or negative control HCCLM3 **(C)**, and Hep3B **(D)** cells treated with the addition of DCN (1 μg/mL) or the negative control. **(E)** Western blot analysis of integrin β1 expression using integrin β1 knocked-down or negative control HCCLM3 and Hep3B cells treated with DCN (1 μg/mL) or the negative control. **(F)** Co-IP assay of DCN with integrin β1, as detected by immunoblot analysis. Scale bars, 100 μm. ITGB1, integrin β1. Data presented as mean ± SEM. *P < 0.05, **P < 0.01, and ***P < 0.001, Student’s *t*-test.

To further confirm that DCN downregulates integrin β1 expression to suppress HCC metastasis, we evaluated the effects of simultaneously modulating DCN and integrin β1 expression in HCC cell lines. The combination of DCN and integrin β1 downregulation further inhibited HCCLM3 and Hep3B cell migration and invasion compared with either DCN or integrin β1 downregulation alone (Figures 7A–D). The expression of integrin β1 was downregulated in each of the DCN and integrin β1 knockdown groups, and further downregulated when DCN was combined with integrin β1 knockdown (Figure 7E and Supplementary Figure 6). These results suggested that DCN binds residual integrin β1 that was not knocked down by shRNA, thus further downregulating integrin β1 to inhibit HCC metastasis. The results of the Co-IP assays using DCN and integrin β1 antibodies showed that integrin β1 was expressed after conjugation with the DCN antibody, which further confirmed direct interaction between DCN and integrin β1 (Figure 7F). Collectively, the combination of DCN and integrin β1 knockdown synergistically augmented the anti-metastatic effects.

## Discussion

Hepatocellular carcinoma is difficult to treat; it recurs at a high rate and metastasizes even after radical surgical resection (Liu et al., 2016). The high propensity of HCC for VI is the main cause of high intrahepatic metastasis (Vilarinho et al., 2017). Both MVI and PVTT are common in VI by HCC, and have become hotspots in studies of HCC prevention and treatment (Wei et al., 2019; Xu et al., 2019). Although clinical strategies such as single surgery, transarterial chemoembolization, targeted, or combined therapies have been applied in attempts to improve therapeutic effects, the clinical benefit for patients with HCC remains poor. Incremental evidence suggests that MVI and PVTT are predictors of poor prognosis for HCC (Forner et al., 2012). However, little is known about the biological molecular mechanisms underlying the evolution of VI. Determining the fundamental events of VI will provide insight for understanding HCC metastasis.

We analyzed the transcriptome of clinical samples from patients who had HCC with or without VI and found that ECM-related pathways are involved in VI by HCC. In addition, DCN secreted by CAFs was downregulated in VI compared with non-VI tissues. Various cell types in the TME, particularly CAFs, play important roles in regulating tumor carcinogenesis and progression. Whether CAF-mediated VI of HCC promotes metastasis remains poorly understood. Consistent with previous findings (Li et al., 2019), DCN was co-expressed with a-SMA but not with E-cadherin, indicating that it is preferentially expressed in fibroblasts and not in epithelial cells. In addition, DCN was gradually downregulated from normal, to primary tumor tissues and even more so in PVTT tissues. These results indicated that fibroblasts in malignant tissues decreased the secretion of DCN to promote VI by HCC, suggesting an anti-metastatic role for DCN secreted by CAFs in HCC. Moreover, we also showed that low DCN expression was associated with a poor prognosis and MVI development. Collectively, these results indicated that DCN secreted by CAFs functions as a tumor suppressor to inhibit VI of HCC.

We analyzed the effects of elevated DCN concentrations in culture medium of HCC cell tumor phenotypes to functionally validate the anti-metastatic role of DCN. Elevated interstitial concentrations of DCN inhibited HCC cell migration and invasion *in vitro*. Decorin functions in the tumorigenesis of various types of cancer (Ju et al., 2015; Reszegi et al., 2020). Delivery of the DCN gene reduced tumor formation in a mouse model of hepatocarcinogenesis evoked by thioacetamide. Serum DCN levels might be associated with the physical function and prognosis of patients with HCC (Kawaguchi et al., 2020). Decorin significantly inhibited the growth potential of various hepatoma cell lines (Horváth et al., 2019). Although these studies found that DCN inhibits the development and growth of HCC, the anti-metastatic role of DCN in HCC has not been determined. To our knowledge, this is the first study to show that DCN secreted by CAFs in the TME is involved in VI by HCC.

We examined ECM pathways that related to cancer progression to identify downstream targets of DCN for promoting tumor metastasis. The expression of integrin β1 was downregulated in cells with elevated DCN, indicating that DCN inhibits HCC metastasis by downregulating integrin β1 expression. Integrin β1 plays crucial roles in cell adhesion, migration, invasion, and proliferation. The role of integrin β1 in tumor growth, tumor recurrence, metastasis and drug resistance is important (Barkan and Chambers, 2011). The expression of integrin β1 in epithelial cells was upregulated in PVTT, compared with tumor and normal tissues. Immunohistochemical co-staining DCN and integrin β1 in the same clinical tissue shows that DCN dynamically regulated the protein expression of integrin β1 in terms of a decrease in DCN accompanied by integrin β1 upregulation from normal, to primary tumor, to PVTT tissues. Its knockdown significantly inhibited HCC cell invasion and migration. Moreover, the combination of DCN and integrin β1 knockdown synergistically augmented the anti-metastatic effects. The results of Co-IP assays showed direct interaction between DCN and integrin β1, thus confirming that DCN-integrin β1 signaling inhibited HCC migration and invasion.

We focused on VI by HCC and identified DCN as a new target for inhibiting HCC intrahepatic metastasis. Our finding that decorin was secreted by fibroblasts indicates that our results offer insight into targeting CAFs in the TME that can be applied to strategies for treating patients who have HCC with PVTT.

## Data Availability Statement

The original contributions presented in the study are included in the article/Supplementary Material, further inquiries can be directed to the corresponding author.

## Ethics Statement

The studies involving human participants were reviewed and approved by Local Ethics Committee of West China Hospital. The patients/participants provided their written informed consent to participate in this study.

## Author Contributions

MX supervised the project, conceived and designed the experiments, analyzed the data, and wrote the manuscript. XZ and PW performed the experiments, analyzed the data, and wrote the manuscript. LiL analyzed the data of IHC staining. JY performed bioinformatics analysis. CY performed the *in vitro* experiments. LX, LiaL, and XC analyzed the data. FD, LF, HZ, and MZ assisted with the writing. All authors contributed to the article and approved the submitted version.

## Conflict of Interest

The authors declare that the research was conducted in the absence of any commercial or financial relationships that could be construed as a potential conflict of interest.

## Publisher’s Note

All claims expressed in this article are solely those of the authors and do not necessarily represent those of their affiliated organizations, or those of the publisher, the editors and the reviewers. Any product that may be evaluated in this article, or claim that may be made by its manufacturer, is not guaranteed or endorsed by the publisher.

## Footnotes

1
http://www.cbioportal.org/

2
https://cancergenome.nih.gov/publications/publicationguidelines

3
https://www.ncbi.nlm.nih.gov/geo/info/disclaimer.html

## Supplementary Material

Supplementary Figure 1 Quantification analysis of DCN expression in normal tissues, tumor tissues and PVTT tissues at protein level.

Supplementary Figure 2 Explore the downstream targets of DCN involved in inhibiting HCC metastasis.

Supplementary Figure 3 Quantification of the WB membranes in Figure 5A.

Supplementary Figure 4 Explore ECM components that could be involved in inhibition of HCC metastasis through DCN upregulation.

Supplementary Figure 5 Expression levels of integrin β1 in clinical matched samples.

Supplementary Figure 6 Quantification of the WB membranes in Figure 7E.
