# Supplementary figures and images for: Cancer-Associated Fibroblasts Promote Vascular Invasion of Hepatocellular Carcinoma via Downregulating Decorin-integrin β1 Signaling
Source: Front Cell Dev Biol. 2021 Aug 24;9:678670. doi: 10.3389/fcell.2021.678670 (PMC8421641; doi:10.3389/fcell.2021.678670)

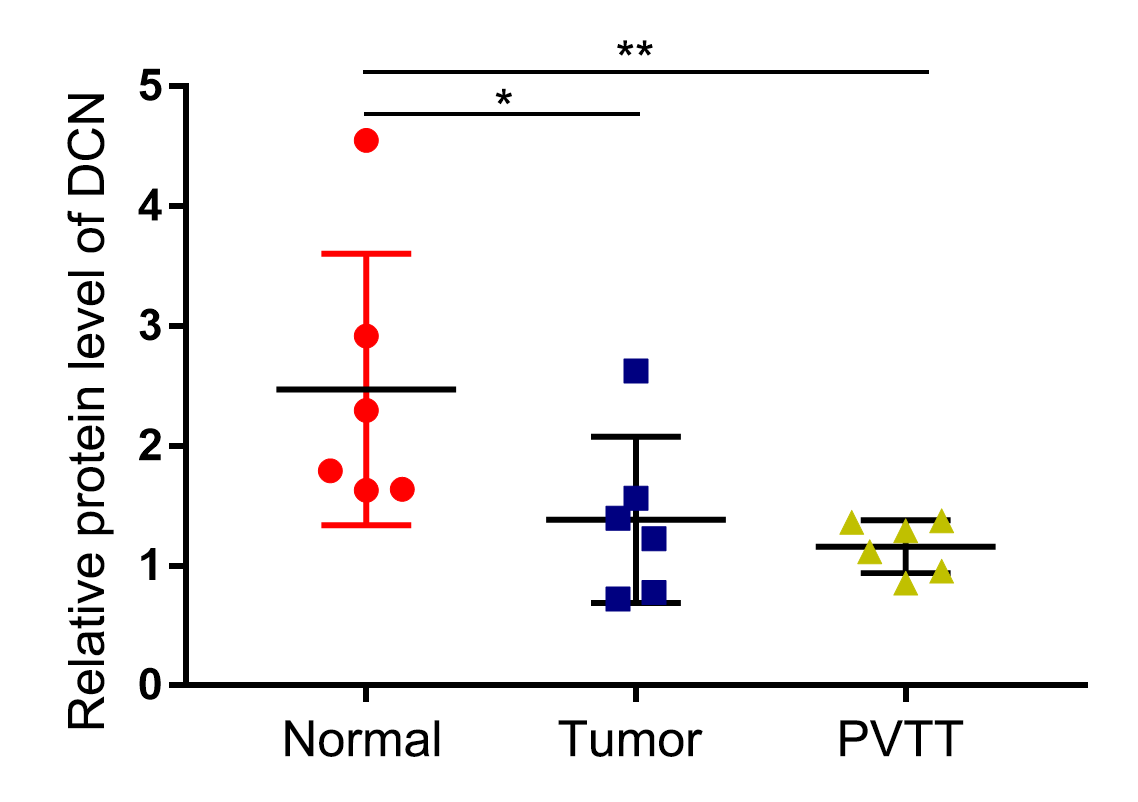

Supplement: Supplementary Figure 1 — Quantification analysis of DCN expression in normal tissues, tumor tissues and PVTT tissues at protein level. [file Image_1.tif]

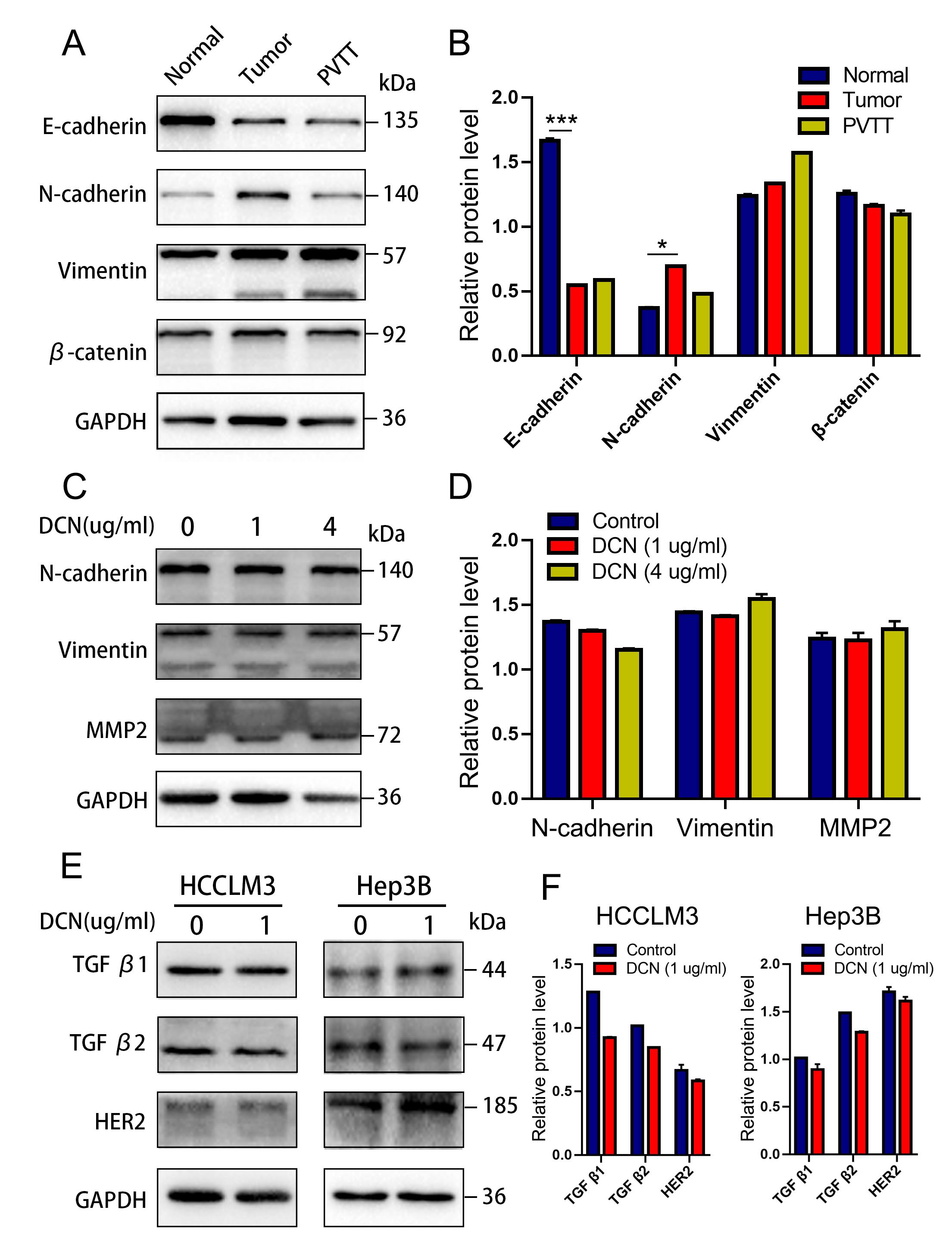

Supplement: Supplementary Figure 2 — Explore the downstream targets of DCN involved in inhibiting HCC metastasis. [file Image_2.tif]

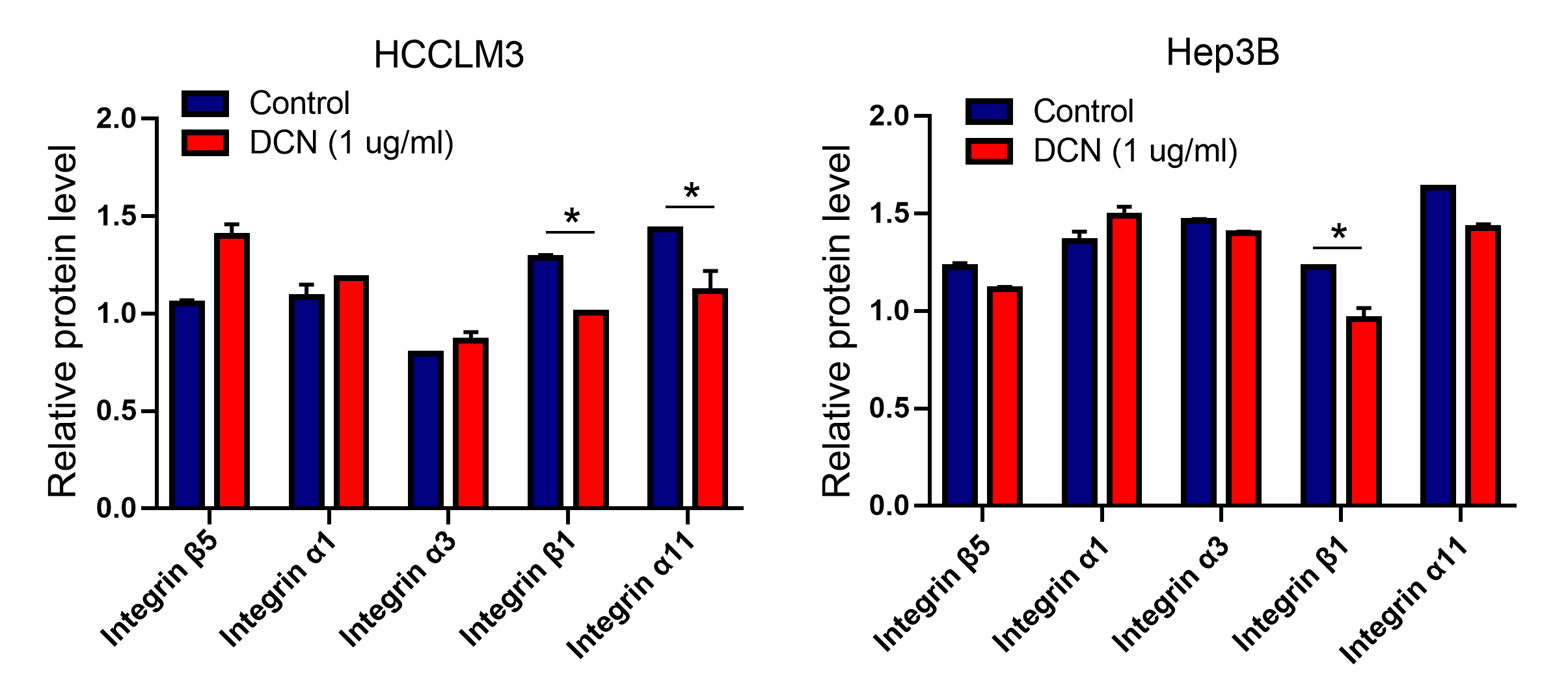

Supplement: Supplementary Figure 3 — Quantification of the WB membranes in Figure 5A. [file Image_3.tif]

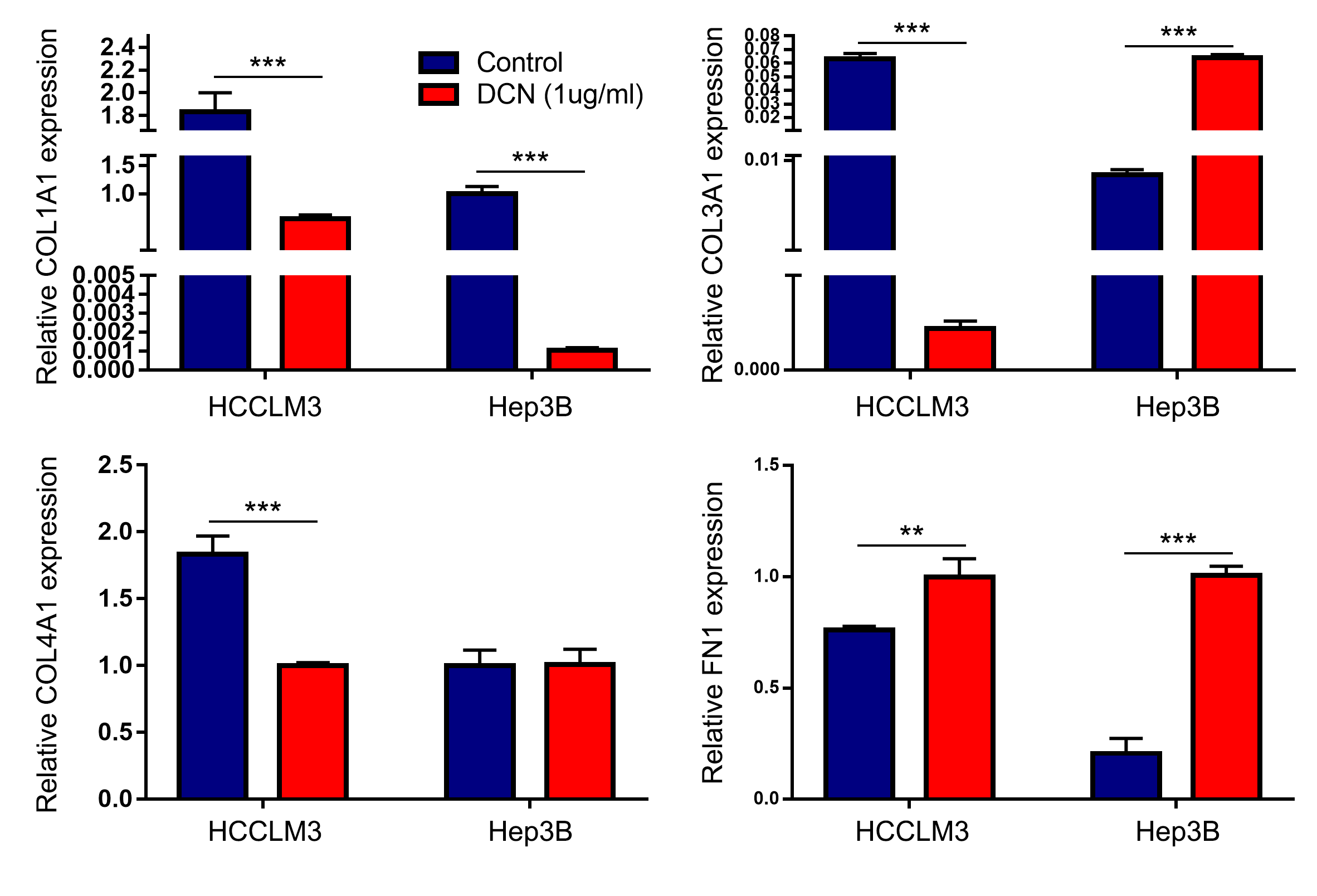

Supplement: Supplementary Figure 4 — Explore ECM components that could be involved in inhibition of HCC metastasis through DCN upregulation. [file Image_4.tif]

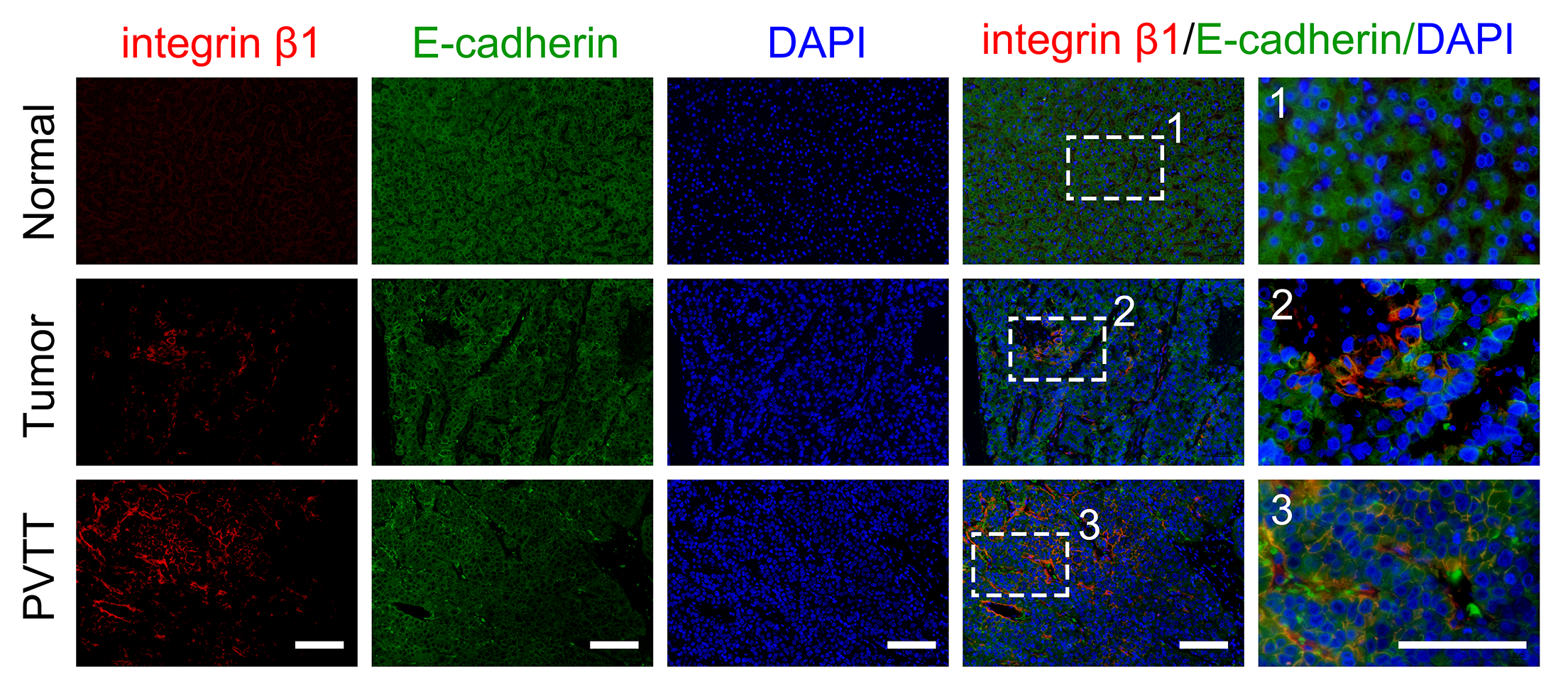

Supplement: Supplementary Figure 5 — Expression levels of integrin β1 in clinical matched samples. [file Image_5.tif]

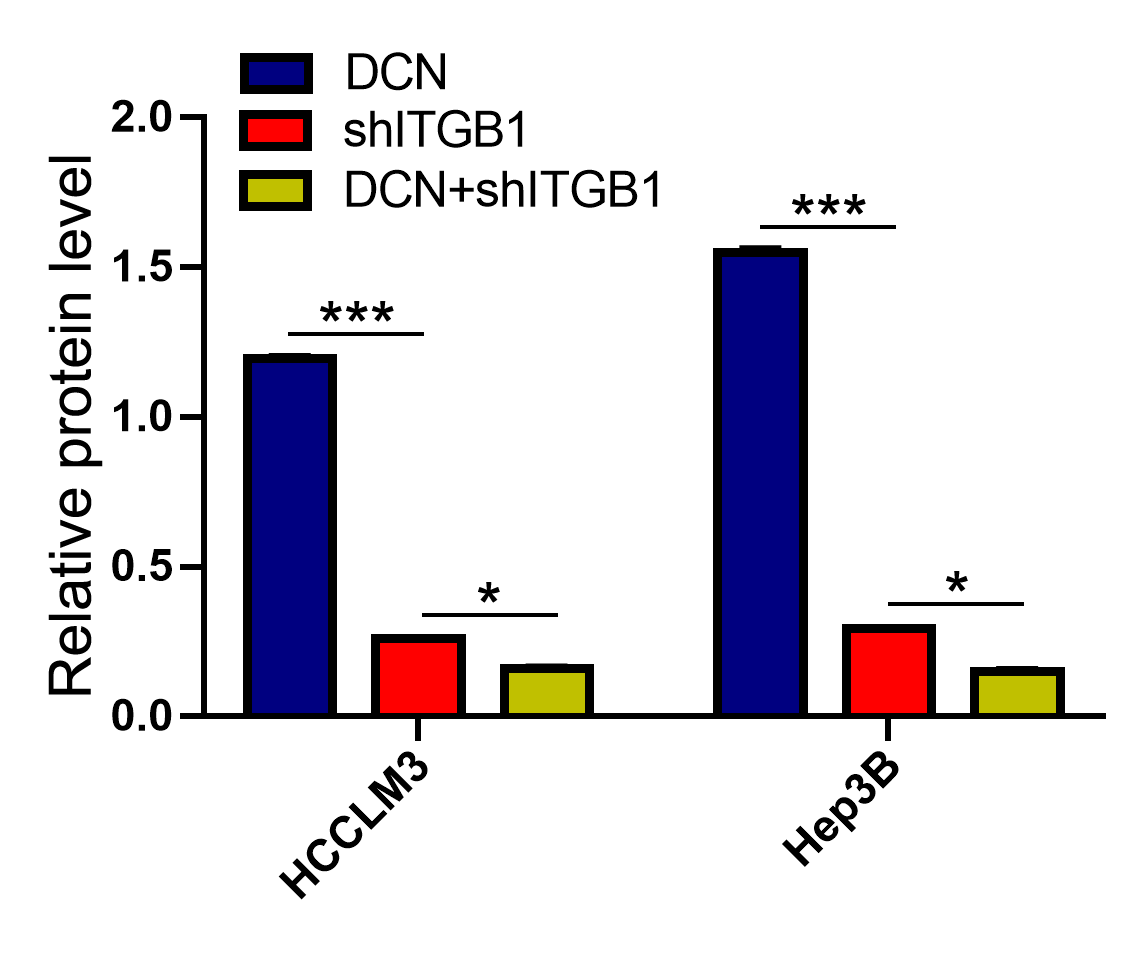

Supplement: Supplementary Figure 6 — Quantification of the WB membranes in Figure 7E. [file Image_6.tif]
